# Spatial heterogeneity in and distributional characteristics of rural ecological livability in China——The case of Fujian Province

**DOI:** 10.1371/journal.pone.0244238

**Published:** 2020-12-29

**Authors:** Xiaoli Jiang, Lingyu Wang, Xiaofeng Su, Weipeng Zeng, Anxin Xu, Qiujin Zheng, Wenxing Xu

**Affiliations:** 1 College of Management, Fujian Agriculture and Forestry University, Fuzhou, Fujian, China; 2 College of Public Management, Fujian Agriculture and Forestry University, Fuzhou, Fujian, China; 3 College of Public Management, The Open University of Fujian, Fuzhou, Fujian, China; 4 Newhuadu Business School, Minjiang University, Fuzhou, Fujian, China; Institute for Advanced Sustainability Studies, GERMANY

## Abstract

With the outbreak of COVID-19, the importance of rural areas has been gradually highlighted, and the importance of rural ecological livability has been gradually recognized. A growing body of literature recognizes the importance of building a rural ecological livability (REL) system. It is urgent that we clarify the status quo and spatial-temporal differences in and distributional characteristics of rural ecological livability and that we carry out targeted and differentiated construction to promote rural ecological livability in post-epidemic China. This study proposes a conceptual model that incorporates various economic, social and environmental factors and develops a comprehensive multifactor (production-living-ecology) evaluation system. Using Fujian Province as an example, the entropy weight method is used to measure the REL level of 55 counties and cities, which are comprehensively evaluated from 2015 to 2019. Moran's I and Getis-Ord Gi* are used to analyze the spatial and distributional characteristics of the REL level in Fujian. The results show that the level of REL in Fujian Province has been relatively flat over the past five years, with a slight downward trend. The overall value of the rural ecological livability index in 2015 was 0.345, and its overall value in 2019 was 0.334, with an average value of 0.343. The REL of Fujian Province is spatially correlated, with high levels of livability in the southeast and low levels in the northeast. The autocorrelation in the level of ecological livability in Fujian's counties and cities continues to increase.

## Introduction

The outbreak of the COVID-19 epidemic has made humankind aware of its own negative ecological impact. To prevent humankind from continuing to excessively invade nature, thereby increasing the number of opportunities for humans to come into contact with different species, including many unknown viruses, it is more urgent than ever to build an ecological civilization in the post-epidemic era. The global epidemic crisis highlights the need for reforms to promote a more transparent and a more resilient ecological process [1]. We must rethink the complex interactions within the ecological balance, considering personal, societal, and ecological health as a system-wide emergent property [2]. More importantly, with the outbreak of COVID-19, the importance of rural areas in ecological reform has been gradually highlighted, and the importance of REL has been gradually recognized [3]. Ecosystem characteristics are vital for the delivery of provisions, regulations, and cultural services [4].

Rural ecology has encountered many problems [5, 6], such as rural nonpoint source pollution [7], heavy-metal pollution of cultivated land and aquatic environments [8], and the dilemma of domestic waste disposal [9], which deserves more attention. The assessment of ecological liability, i.e., ecological vulnerability, is critical for research on ecological change [10, 11]. Rural ecology is directly related to ecological security. Authorities have called for solid efforts to improve rural living environments in China. Consistent efforts such as the rural "Toilet Revolution" as well as sewage and garbage treatment are being made to improve rural living environments and to preserve the beautiful scenery in Chinese villages. With the implementation of the Chinese rural revitalization strategy, ecological livability has become a hot research topic in rural development, and the concept of livability includes the country's new ideas and grand blueprint for rural construction. Because ecological resources are valuable, it is necessary to reconstruct the REL evaluation system in the post-epidemic era.

In 1961, the World Health Organization proposed four basic concepts that define the living environment: safety, health, convenience and comfort. Ebenezer [12] proposed the idea of a livable city in view of urban environmental pollution, population expansion and other issues, including housing comfort, convenient transportation and environmental health. Casellati [13] and Lennard [14] claimed that urban livability needs to ensure comfortable living for people and ecological sustainability, as well as a sense of the reality of the living space. Evans [15] defined livability as livelihood and ecological sustainability. The former includes living conditions, the work environment, income and the comfort level of public facilities and services, while the latter includes good resources such as water and soil. In recent years, scholars have adopted livability measures that rely on performance metrics for transportation choices, land use, economic prosperity [16], cultural vitality, and education [17].

To evaluate the livability of cities, some scholars use combinations of subjective and objective measures to establish an evaluation index system [18], while others use multivariate statistical analysis [19] and GIS spatial analysis technology to evaluate the city's livability level [20].

However, there is still no unified definition and measurement of livability in the literature due to its comprehensive and dynamic meaning. For instance, livability has been defined as suitability for human living [21] and the quality of life experienced by the residents of a city or region [22]. It has also been defined using six principles of livability incorporated by the US Department of Housing and Urban Development (HUD) and the US Environmental Protection Agency (EPA) [23].

In this paper, we define livability as an ecology that is reflective of the highly coupled relationship between natural ecology and human ecology. Livability is the organic unity of human nature's pursuit of health and of pleasure. Ecological livability is the combination of both rural ecology and rural livability, rather than merely a reference to the ecological aspects. The core elements of rural ecological livability include the livability of the rural production environment, the livability of the living environment and the livability of the ecological environment. The majority of previous studies focus on evaluating urban ecological livability rather than rural livability. Furthermore, while these evaluation indices have merits, they also have fundamental problems, such as discussing only ecology rather than depicting a comprehensive vision of rural life.

Most studies mainly focus on urban ecological livability in the context of temporal trends but rarely take rural areas into consideration to explore spatial heterogeneity in ecological livability there. China's government has proposed the formation of a reasonable structure for productive, living and ecological space in accordance with the overall requirements of promoting intensive and efficient production space, the moderate livability of living spaces, and beautiful scenery in ecological spaces. It emphasizes the concept of ecological space and the spatial unity of production-living-ecology, especially during the COVID-19 epidemic.

Our study contributes to the literature in a number of ways. Since the previous literature offers little guidance on REL, our first contribution is to draw upon the preceding literature to propose a more realistic evaluation index system for integrating economic analyses within ecosystem service assessments. Second, we propose a conceptual model to assess REL that extends the concept of livability by including a component related to environmental sustainability and visualizes the spatial-temporal transformation of REL in Fujian Province based on the LISA cluster diagram. Third, methodologically, the entropy weight method helps to avoid subjectivity when assigning weights. Moran's I and Getis-Ord Gi* are used to analyze the spatial and temporal differences in and distributional characteristics of the REL level in Fujian Province.

The rest of the paper is organized as follows. The next section describes an integrated multi-factor (production-living-ecology) assessment model and establishes ecological livability evaluation indicators, then presents the study area, data sources and methods used in this study. The analysis of the results and recommendations are given in Section 3. The final section presents the conclusions and a discussion. This paper clarifies the spatial heterogeneity of REL in Fujian Province and has important implications for China's ecological protection and rural development strategy.

## Materials and methods

### Construction of an evaluation index system

Livability is a multidimensional concept that includes the economy, the environment, life and society. As a whole system, these elements are sustainable, long-lasting and constant. This is the concept of livability pursued by rural villages, which encompasses the core concept of a livable city. Regarding the understanding of ecological-human interactions, it is believed that human activities have an important impact on REL. The assessment of ecological livability is essential for managing and ensuring eco-environmental stability.

Suitability evaluation analysis is a method that pursues a dynamic balance among multiple elements, scales and functions. Rural function is a comprehensive and complex concept that includes economic development, food production, ecological conservation and social security. A countryside that is beautiful and pleasant to live in and ecologically livable is not only ecologically sound but is also a complex combination of rural economic development, affluent living and good ecology.

**Fig 1:**
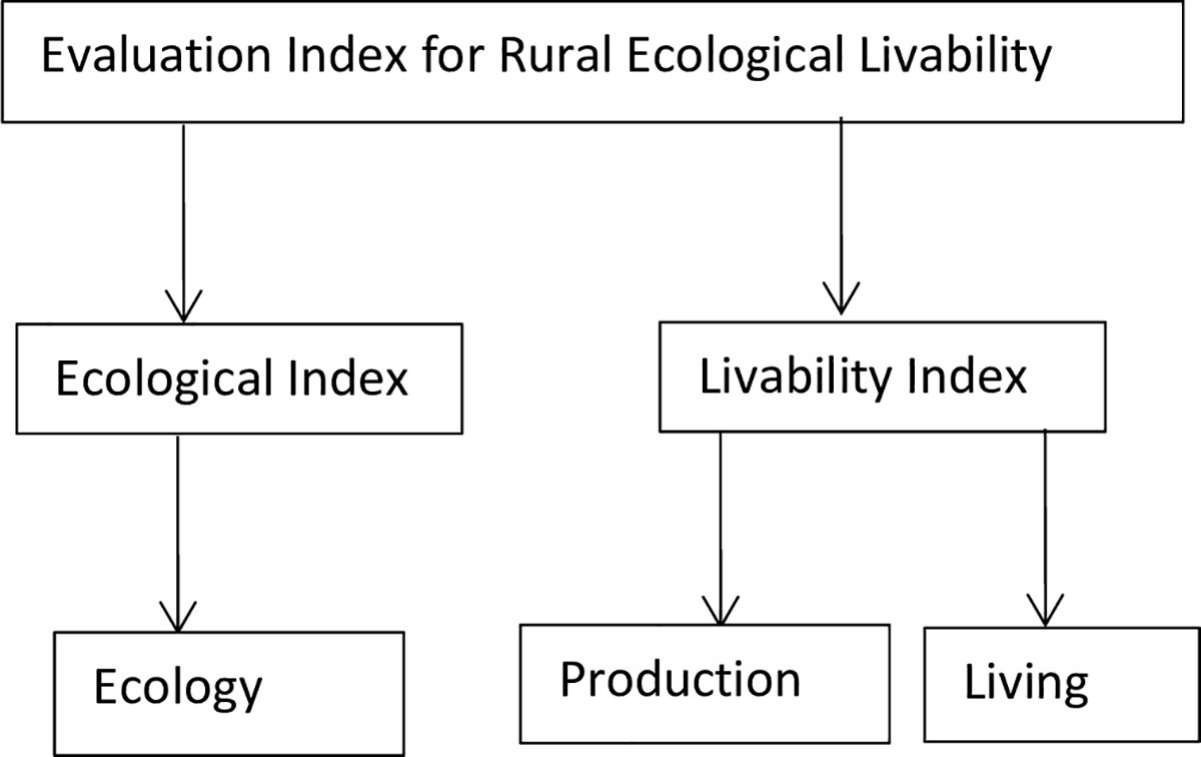
Content of ecological livability evaluation.

Especially in consideration of the crisis brought about by COVID-19, villages should be the foundation of China's economic development. The ecological livability of rural areas not only refers to a good ecological environment but also includes the ability to live self-sufficiently under special circumstances. An ideal evaluation model for REL should consider the effects (positive or negative) of production, living and ecology. Ecological livability also implies that quality of life is created by an ongoing interaction of community, environmental and economic qualities [24]. We believe that the rural environment and rural resources have the functions and attributes of production, living and ecology (shown in Fig 1).

### Explanation of the evaluation index system

Rural settlements are residential areas in rural environments where the population has settled based on a close relationship with agriculture in both geography and function. Rural life-quality is a multidimensional concept used to measure citizen living standards and states and which incorporates the economy, society, culture, politics, and ecology and includes both material and nonphysical components. Multifunctional zoning is the foremost basis for developing differentiated spatial planning systems and management policies for territorial spaces.

The evaluation of REL needs to take into account that the characteristics of ecological resources and spatial resources (landscapes, forests, lakes and grass) are living communities, and these concepts are inseparable or sticky. Productive-living-ecological space includes intensive and efficient production space, livable and appropriate living space, and beautiful ecological space (see Table 1). "Production-living-ecology appropriate" is a beautiful rural environment suitable for living, working and traveling.

**Table 1:** Rural ecological livability evaluation index system.

Goal Layer	Criterion Layer	Factor Layer
Evaluation of Rural Ecological Livability in Fujian Province	Quality of production development	X11: Per-capita GDP (Ten thousand yuan)
		X12: Proportion of income from special industries (%)
		X13: Total mechanical power per unit of cultivated land (W/mu)
		X14: Total grain output (Tons)
		X15: Value-added of secondary industry (Ten thousand yuan)
		X16: Agriculture, forestry and water affairs expenditure (Ten thousand yuan)
		X17: Annual financial income per capita (Yuan)
	Quality of living environment development	X21: Per-capita net income of farmers (Yuan)
		X22: Rate of decline of the number of rural residents with minimum living security (%)
		X23: Highway density (Km)
		X24: Number of high school teachers (per 1,000 students)
		X25: Number of beds in medical and health institutions
		X26: Completed investment in fixed assets of rural households (Ten thousand yuan)
		X27: Urbanization rate (%)
	Quality of ecological development	X31: Fertilizer application intensity (Tons)
		X32: Air quality index
		X33: Days of air compliance (%)
		X34: Total area of afforestation (Mu)
		X35: Forest coverage rate (%)

### Study area

Fujian Province is located on the southeast coast of China. The study area is situated at 23°30'–28°20' N and 115°40'–120°30' E, which has a subtropical maritime monsoon climate. The annual average temperature is approximately 15–22°C. The total land area is 121,400 square kilometers. The terrain is high in the northwest and low in the southeast, which are near the mountains and the sea, respectively. The mountainous and hilly areas within the territory account for approximately 90% of the total area of the province.

Fujian Province is the first province in China to propose an ecological province strategic concept. In 2014, it was approved as the country’s first provincial-level demonstration zone of ecological civilization pilot, and in 2016 it became the first national pilot zone for ecological civilization. Its explorations into reform and innovation need to be summarized and reported. In terms of green development, Fujian Province has a faster pace than other provinces. Therefore, it is necessary to analyze changes in the REL of the experimental area to provide corresponding policy recommendations for subsequent ecological development.

### Data sources

Because our research subject is rural areas in Fujian Province, the main data come from the 2015–2019 Fujian Provincial Statistical Yearbook (http://tjj.fujian.gov.cn/xxgk/ndsj/), China County Statistical Yearbook from 2015 to 2019 (https://data.cnki.net/trade/Yearbook/Single/N2016030043?z=Z001), the City of Fujian Statistical Yearbook, and Fujian County (City) National Economic and Social Development Statistical Reports. Due to the lag in statistics, it is generally released one year later. Therefore, the 2015–2019 statistical yearbook reflects the development of 2014–2018. In order to discuss consistency, this article adopts the former caliber. Due to a lack of ecological data for the county area, this paper uses the virtual value method based on the growth trend of the time series to supplement the missing air quality data and number of days of air compliance in each county from 2014 to 2015.

### Study methods

#### Standardization of indicators

First, to eliminate differences in the indices, variables are standardized. Let X_ijn_ be the j-th index of the i-th region in year n, and Y_ijn_ be the standardized index value corresponding to X_ijn_.

The guiding standardization formula is as follows.

$$Y_{ijn}=\frac{X_{ijn}-i_{min}n_{min}\left(X_{ijn}\right)}{i_{max}n_{max}\left(X_{ijn}\right)-i_{min}n_{min\left(X_{ijn}\right)}}$$ (1)

The binding standardization formula is as follows.

$$Y_{ijn}=\frac{i_{max}n_{max}\left(X_{ijn}\right)-X_{ijn}}{i_{max}n_{max}\left(X_{ijn}\right)-i_{min}n_{min\left(X_{ijn}\right)}}$$ (2)

#### Entropy method

The entropy method [25] uses the information inherent in the indicator to judge the utility value of the indicator and avoids bias caused by subjective factors to a certain extent. Due to the advantages of both objective index weighting and a transparent system structure, this paper provides an effective tool for the measurement and analysis of REL.

Information entropy is a measure of uncertainty and of the degree of dispersion. The greater the degree of dispersion in the indicators, the greater is that indicator's contribution to the evaluation system and the greater is its weight. Considering that the weights of the indicators differ in each year, this study separately calculates the information entropy of each indicator in each year of the study to obtain the weights of the indicators for each year. Since the calculation of information entropy involves the natural logarithm, the entropy method requires the fuzzy comprehensive evaluation matrix to include only real numbers greater than 0. Because the lowest normalized value of each indicator is 0, all the standardized values of the indicator are shifted to the right by one unit before weighting the indicator. Table 2 uses the entropy weight method to obtain the index weights of the REL measures for each county in Fujian Province from 2015 to 2019. Finally, on the basis of the index weights, the comprehensive REL score of each city and county in Fujian Province is calculated.

**Table 2:** Weight of criterion layer and index layer.

Factor	2015	2016	2017	2018	2019	Max Value	Min Value	Mean Value	Standard Deviation
*X11*	0.0341	0.0342	0.0299	0.0365	0.0190	0.0342	0.0190	0.0336	0.0028
*X12*	0.0005	0.0005	0.0004	0.0003	0.0002	0.0005	0.0002	0.0004	0.0001
*X13*	0.0576	0.0613	0.0539	0.0693	0.0401	0.0693	0.0401	0.0605	0.0066
*X14*	0.0678	0.0724	0.0023	0.0891	0.0409	0.0891	0.0023	0.0579	0.0382
*X15*	0.1971	0.2008	0.1697	0.1958	0.1015	0.2008	0.1015	0.1908	0.0143
*X16*	0.0274	0.0281	0.0264	0.0283	0.4212	0.4212	0.0264	0.0276	0.0009
*X17*	0.1018	0.0983	0.1046	0.0991	0.0431	0.1046	0.0431	0.1010	0.0029
*X1*	0.4863	0.4955	0.3871	0.5184	0.6661	0.6661	0.3871	0.4718	0.0581
*X21*	0.0043	0.0044	0.0039	0.0043	0.0022	0.0044	0.0022	0.0042	0.0002
*X22*	0.0191	0.0218	0.0145	0.0109	0.0045	0.0218	0.0045	0.0166	0.0048
*X23*	0.0378	0.0381	0.0323	0.0362	0.0186	0.0381	0.0186	0.0361	0.0027
*X24*	0.0911	0.0931	0.0806	0.0845	0.0474	0.0931	0.0474	0.0873	0.0058
*X25*	0.0133	0.0147	0.0126	0.0155	0.0058	0.0155	0.0058	0.0140	0.0013
*X26*	0.0998	0.1050	0.1517	0.0962	0.1295	0.1517	0.0962	0.1132	0.0260
*X27*	0.0007	0.0007	0.0005	0.0006	0.0003	0.0007	0.0003	0.0006	0.0001
*X2*	0.2660	0.2778	0.2962	0.2482	0.2083	0.2962	0.2083	0.2720	0.0202
*X31*	0.1343	0.1358	0.1198	0.1333	0.0854	0.1358	0.0854	0.1308	0.0074
*X32*	0.0007	0.0010	0.0080	0.0060	0.0012	0.0080	0.0007	0.0039	0.0036
*X33*	0.0000	0.0000	0.0066	0.0001	0.0001	0.0066	0.0000	0.0017	0.0033
*X34*	0.1107	0.0881	0.1806	0.0919	0.0380	0.1806	0.0380	0.1179	0.0430
*X35*	0.0020	0.0017	0.0016	0.0022	0.0009	0.0022	0.0009	0.0019	0.0002
*X3*	0.2477	0.2266	0.3167	0.2335	0.1256	0.3167	0.1256	0.2561	0.0413

#### Moran's I

Moran's I is used to measure the degree of global autocorrelation in REL for Fujian Province, and local autocorrelation and spatial agglomerations are analyzed using a Moran's I scatter plot and LISA cluster map.

This paper uses the global Moran's I to analyze the spatial correlations among rural livability levels in Fujian Province. Moran's I ranges between [–1, 1]. If Moran's I is closer to -1, it indicates that the livability levels exhibit significant spatial differences. If Moran's I tends towards 1, it means that there is lear spatial agglomeration, and the differences in livability levels between a given region and its surrounding regions are small.

$$\mathrm{Moran}’s\;I=\frac{\sum_{i=1}^{n}\sum_{j=1}^{n}w_{ij}\left(x_{i}-\bar{x}\right)\left(x_{j}-\bar{x}\right)}{S^{2}\sum_{i=1}^{n}\sum_{j=1}^{n}w_{ij}}$$ (3)

where $S^{2}=\sum_{i=1}^{n}(x_{i}-\bar{x})$ and $\bar{x}=1/\sum_{i=1}^{n}x_{i}$; n represents the number of counties (cities) in Fujian Province; w_ij_ denotes the spatial weight matrix; x_i_ denotes the comprehensive ecological livability score for each county (city); and $\bar{x}$ denotes the average comprehensive ecological livability score for each county (city).

## Results and discussion

### Comprehensive REL score

The comprehensive measurement of and ranking report for REL in Fujian Province from 2015 to 2019 is shown in Table 3. The counties and cities with higher REL are Jinjiang, Nan'an, Anxi, Fuqing, and Longhai. The level of REL in Fujian Province exhibits a fluctuating downward trend over the past five years. Its overall value was 0.345 in 2015 and 0.334 in 2019. The average value is 0.343. Among the 55 counties and cities in Fujian Province, those whose REL improved significantly over the five years studied are Fuding (19.39%), Fuqing (14.204%), and Anxi (13.368%). The cities with significant declines are Shouning (-27.801%), Shunchang (-17.79%), and Zhangpu (-16.617%).

**Table 3:** Comprehensive measurement results and ranking of REL in Fujian Province from 2015 to 2019.

City/County	2015	2016	2017	2018	2019	Average Value	Rank
*Fuqing*	0.4931	0.4844	0.5012	0.4678	0.5747	0.5042	4
*Minhou*	0.4160	0.4568	0.4827	0.4348	0.3797	0.4340	7
*Lianjiang*	0.3750	0.3696	0.3665	0.3971	0.3526	0.3722	18
*Luoyuan*	0.2705	0.2713	0.2694	0.3124	0.3041	0.2855	41
*Minqing*	0.2893	0.2720	0.2802	0.2898	0.2915	0.2846	42
*Yongtai*	0.2484	0.2762	0.2793	0.2710	0.2738	0.2697	45
*Pingtan*	0.3081	0.3222	0.3342	0.3135	0.2784	0.3113	33
*Xianyou*	0.4220	0.4455	0.4231	0.4321	0.4061	0.4258	8
*Yongan*	0.3655	0.3809	0.4574	0.3889	0.4116	0.4009	12
*Mingxi*	0.2740	0.2455	0.2557	0.2577	0.2775	0.2621	46
*Qingliu*	0.2662	0.2623	0.2589	0.2667	0.2550	0.2618	47
*Ninghua*	0.3271	0.3085	0.2866	0.3050	0.3034	0.3061	34
*Datian*	0.2955	0.3133	0.2813	0.2995	0.2982	0.2976	35
*Youxi*	0.3899	0.3609	0.3260	0.3583	0.3157	0.3501	22
*Shaxian*	0.3265	0.3233	0.2935	0.3114	0.3124	0.3134	31
*Jiangle*	0.2906	0.2738	0.2571	0.2676	0.2849	0.2748	44
*Taining*	0.2516	0.2544	0.2434	0.2404	0.2467	0.2473	49
*Jianning*	0.2584	0.2534	0.2465	0.2540	0.2502	0.2525	48
*Shishi*	0.4565	0.4656	0.4824	0.4522	0.4472	0.4608	6
*Jinjiang*	0.6486	0.6442	0.6764	0.6764	0.6424	0.6576	1
*Nan'an*	0.5319	0.5217	0.5109	0.5224	0.4997	0.5173	2
*Hui'an*	0.4137	0.4132	0.4284	0.4125	0.4572	0.4250	9
*Anxi*	0.4956	0.4970	0.4900	0.5110	0.5721	0.5132	3
*Yongchun*	0.3893	0.3598	0.3465	0.3709	0.3713	0.3675	21
*Dehua*	0.3063	0.3161	0.3481	0.3093	0.3034	0.3166	29
*Longhai*	0.4711	0.4768	0.5039	0.4944	0.4692	0.4831	5
*Yunxiao*	0.2950	0.3031	0.3017	0.3146	0.2659	0.2961	36
*Zhangpu*	0.4450	0.4299	0.4025	0.3921	0.3816	0.4102	10
*Zhaoan*	0.3244	0.3271	0.3246	0.3335	0.3015	0.3223	28
*Changtai*	0.3472	0.3445	0.3398	0.3403	0.3235	0.3391	25
*Dongshan*	0.2976	0.3001	0.3124	0.2895	0.2626	0.2924	39
*Nanjing*	0.4000	0.3887	0.4030	0.3984	0.3771	0.3934	14
*Pinghe*	0.3941	0.4007	0.4120	0.4056	0.3829	0.3991	13
*Hua’an*	0.3040	0.2731	0.2919	0.2794	0.2974	0.2892	40
*Shaowu*	0.4179	0.3799	0.3683	0.3851	0.3616	0.3825	17
*Wuyishan*	0.3555	0.3290	0.3208	0.3275	0.3086	0.3283	27
*Jianou*	0.3897	0.4104	0.4275	0.4393	0.3776	0.4089	11
*Shunchang*	0.3056	0.2758	0.2713	0.2729	0.2594	0.2770	43
*Pucheng*	0.3624	0.3512	0.3276	0.3618	0.3309	0.3468	23
*Guangze*	0.2845	0.3015	0.3213	0.3089	0.2471	0.2927	37
*Songxi*	0.2412	0.2357	0.2325	0.2377	0.2303	0.2355	53
*Zhenghe*	0.2457	0.2514	0.2467	0.2398	0.2368	0.2441	51
*Zhangping*	0.3203	0.3170	0.4140	0.3173	0.3059	0.3349	26
*Changding*	0.3688	0.3829	0.3737	0.3729	0.3586	0.3714	19
*Shanghang*	0.3713	0.4170	0.3823	0.3713	0.3787	0.3841	16
*Wuping*	0.3291	0.3637	0.3538	0.3384	0.3343	0.3439	24
*Lianping*	0.3229	0.3345	0.3023	0.3080	0.3006	0.3136	30
*Fu’an*	0.3766	0.3972	0.3791	0.3992	0.3699	0.3844	15
*Fuding*	0.3458	0.3465	0.3484	0.3747	0.4289	0.3688	20
*Xiapu*	0.3102	0.3264	0.3061	0.3292	0.2884	0.3121	32
*Gutian*	0.3018	0.3006	0.2838	0.3122	0.2646	0.2926	38
*Pingnan*	0.2290	0.2494	0.2356	0.2491	0.2213	0.2369	52
*Shouning*	0.2805	0.2379	0.2389	0.2454	0.2195	0.2444	50
*Zhouning*	0.2255	0.2157	0.2248	0.2167	0.1981	0.2162	55
*Zherong*	0.2276	0.2405	0.2239	0.2218	0.2147	0.2257	54

The top ten ranked cities and counties are Jinjiang (0.6576), Nan'an (0.5173), Anxi (0.5132), Fuqing (0.5042), Longhai (0.4831), Shishi (0.4608), Minhou (0.4340), Xianyou (0.4258), Hui'an (0.4250) and Zhangpu (0.4102).

The development of the core REL subsystems in some counties is unbalanced. The areas with better development of production livability are concentrated in the southeast of Fujian, while development in the northwestern area is relatively weak; the areas with better development of living environment livability are concentrated in the southeast of Fujian, while the development in the northwest is relatively weak; and in terms of ecological livability, the northwestern area, which has the inherent advantages of a "green treasury" and a "southern forest sea" but lags behind in the development of production and living environment livability, has the highest ecological environment score, while the southern area, in which productive and living environment livability are better developed, has a lower ecology score. To achieve a high REL level, we must pay coordinate the development of the three core elements of production, ecology and living instead of neglecting one of them.

Based on the REL levels of the counties in Fujian Province from 2015 to 2019, the area is divided into the following four categories.

The first category includes cities and counties in which the level of ecological livability is above the average level for all counties in Fujian, and the level of ecological livability lags behind that of production and living environment livability. These counties include Fuqing, Jinjiang and others. In the early stage of rural development, excessive emphasis was paid to rural production functions, which resulted in the deterioration of parts of the rural ecological environment. In particular, the excessive use of pesticides, fertilizers, and plastic films in agricultural production has caused nonpoint source pollution problems, which have led to a series of ecological problems, such as water eutrophication and soil degradation.

With the slowdown of economic development, the ecological problems caused by agricultural construction have received increasing attention. Through the formulation of rural residential-environment planning, which focuses on rural environmental improvement and the recovery of rural agricultural production pollutants and encourages the development of ecological and green modern agriculture, rural "production-ecology" coupling has been significantly improved.

The second category includes the cities and counties in which the level of ecological livability is above the average level for all counties in Fujian, and the production and living environment livability levels lag behind the ecological livability level. These counties and cities include Minhou, Zhangpu and others. Due to the small size of these counties and cities and relatively inferior geographical location, there is a relatively low level of economic development. Such counties and cities can take advantage of ecological livability, promote ECO development, and transform ecological advantages into economic advantages.

The third category includes counties and cities in which the level of ecological livability lags behind its average level for all counties in Fujian, and the level of ecological livability lags behind that of the production and living livability level. These include Datian, Changtai and others. Such counties and cities are mainly located in mountainous areas, and their living conditions have declined. Such cities should improve their public infrastructure facilities while improving their development and employment environments and encouraging low-carbon activities.

The fourth category includes counties and cities in which the overall level of REL lags behind the average REL level of all counties in Fujian, and the livability of production and living lags behind the construction of ecological livability. These counties include Zhenghe and Zhouning. This type of area should improve production and living capacity, with livable production as the main development goal.

Analyzing the factors that influence REL in Fujian Province and the index layer levels shows that the value-added of the secondary industry and the per-capita annual fiscal revenue in each region are important drivers of REL, while the amount of chemical fertilizer applied and reductions in the ecological afforestation area are important reasons leading to the decline of the REL level.

### Spatial correlation of REL in Fujian Province

#### Global autocorrelation analysis

The agglomeration and dispersion effects in the ecologically livable horizontal space are tested using Moran's I. Table 4 reports the global Moran's I for the REL levels in the counties and cities of Fujian Province from 2015 to 2019. The overall trend is an increase from 0.3697 in 2015 to 0.5362 in 2019, with an average value of 0.4446, showing a positive correlation. The P-values of the global Moran's I are 0.0010, 0.0009, 0.0012, 0.0007, and 0.0005. The ecological livability of all counties and cities in Fujian showed significant autocorrelation, and the Z-test showed that the spatial correlation gradually increased. In general, autocorrelation in the REL level in counties and cities in Fujian continues to increase, and there is a significant increase in spatial-temporal correlation, which indicates the existence of a spatial agglomeration effect.

**Table 4:** Global Moran's I.

Year	2015	2016	2017	2018	2019	Average Value
*Moran's I*	0.3697	0.3878	0.4161	0.5131	0.5362	0.4446
*Variance*	0.0641	0.0642	0.0615	0.0616	0.0611	0.0625
*Z Score*	6.1445	6.7871	7.3305	7.5103	7.7649	7.1075
*P value*	0.0010	0.0009	0.0012	0.0007	0.0005	0.0009

#### LISA cluster diagram

**Fig 2:**
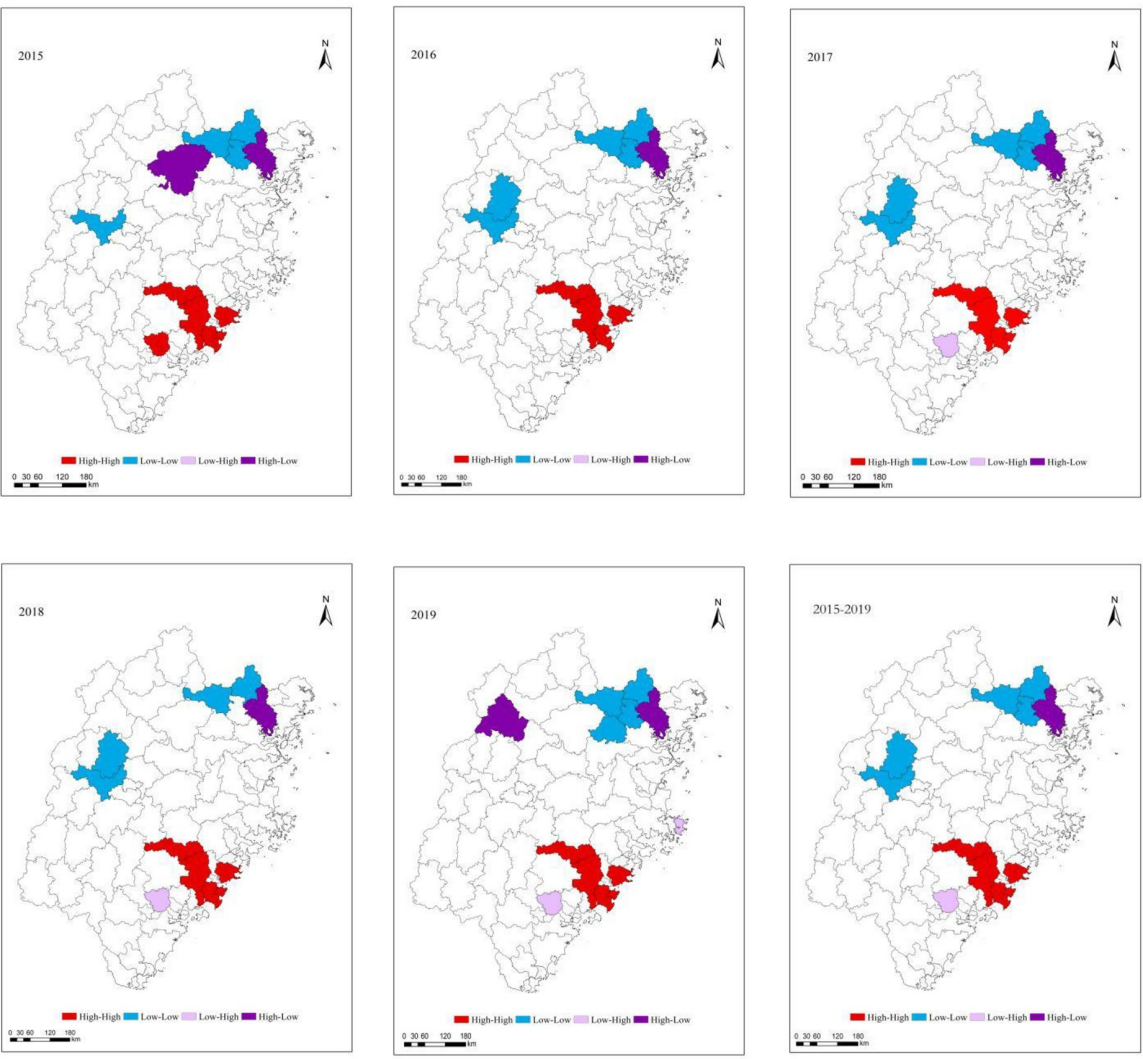
LISA cluster diagram of REL in Fujian Province from 2015 to 2019. (Source of base map: The open source map data service provided by the National Platform for Common GeoSpatial Information Services (https://www.tianditu.gov.cn/)).

Further, Fig 2 shows the evolution of REL in the horizontal space of various counties and cities in Fujian Province from 2015 to 2019. The results show an obvious overall high-high agglomeration, and the low-low agglomeration is also obvious. The REL of Fujian Province tends to be spatially higher on the southeast coast and lower inland in the northeast.

*High-high agglomeration areas (H-H)*. The high-high agglomeration areas, including Hui'an, Fuqing, and Fuding, are mainly concentrated in the southeast coastal area. There is a trend towards agglomeration clusters from Dehua to Jinjiang. But Dehua changed from High-high to Low-high in 2019. The county regions and adjacent areas with high-high agglomeration have relatively high levels of ecological livability and good diffusion effects.

*Low-low agglomeration areas (L-L)*. Most low-low agglomeration areas are located in the northeast. The REL of this type is low, and the surrounding areas also have a low level of ecological livability, resulting in low-low agglomeration. Zhouning and Shouning are the first low-low agglomeration areas, which gradually come to include Mingxi and Jiangle in 2016. In 2019, Mingxi and Jiangle are no longer Low-low. The low-low agglomeration area develops from dot-shaped to cluster-shaped. Although the level of production livability in these areas is not high, their ecological resources are relatively high, and they have the potential to develop and improve their REL, which would play a positive role in improving the overall REL in Fujian Province.

*High-low agglomeration areas (H-L)*. The high-low agglomeration areas mainly include Fu'an and Jianou, which are between the low-low agglomeration areas and the high-high agglomeration areas. Given the proliferation of the high-high agglomeration areas, Jianou are likely to develop further.

*Low-high agglomeration areas (L-H)*. Low-high agglomeration is not significant. The low-high agglomeration areas are scattered in the central mountainous area. Typical representatives of this type are Changtai.

#### Spatial pattern of cold and hot distribution of REL in Fujian Province

**Fig 3:**
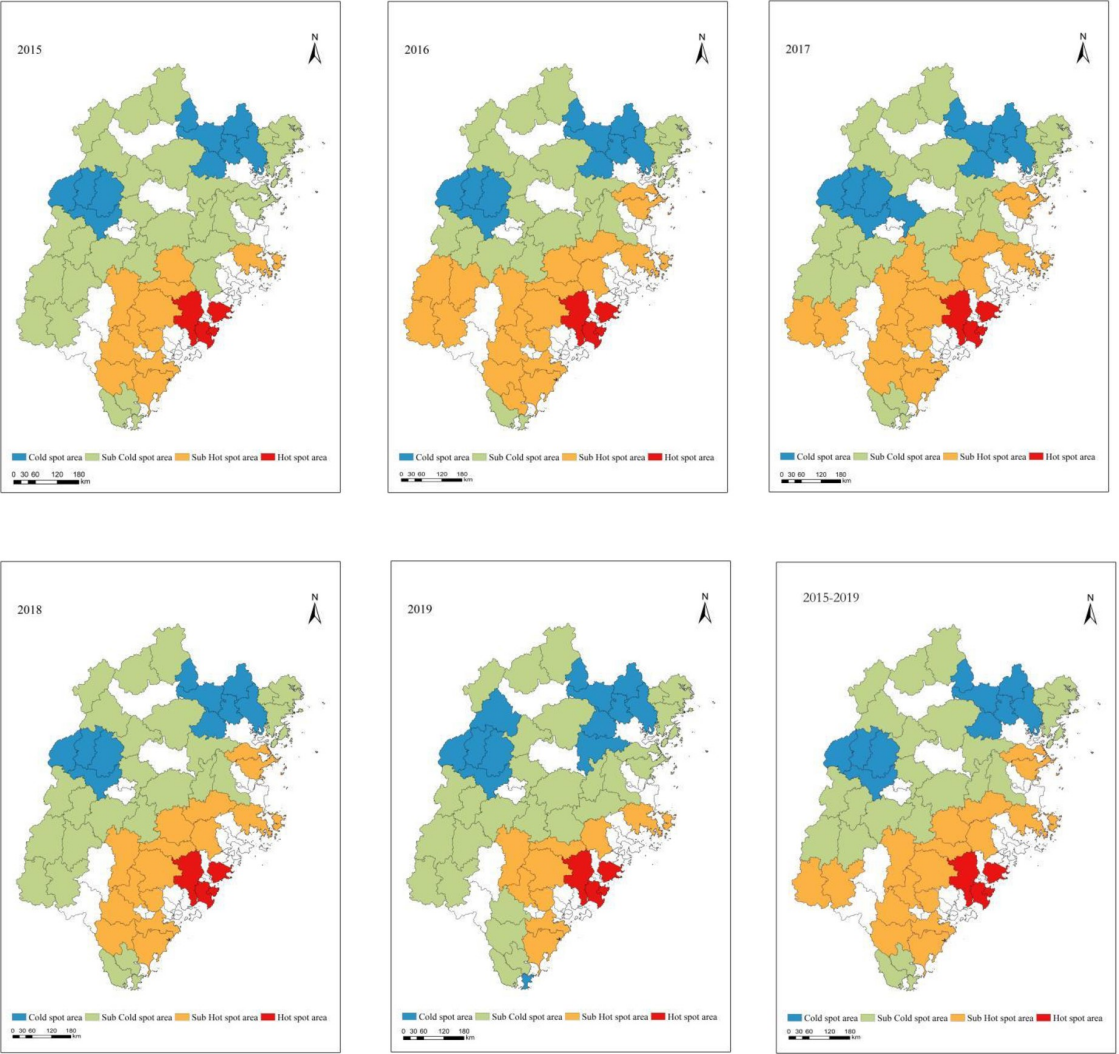
G index spatial distribution of REL in Fujian Province from 2015 to 2019. (Source of base map: The open source map data service provided by the National Platform for Common GeoSpatial Information Services (https://www.tianditu.gov.cn/)).

The spatial models that estimate the cold and hot spots of spatial aggregation are calculated by Getis-Ord Gi*. Getis-Ord Gi* is a spatial statistical method that can describe and create visualizations of spatial distributions, find local spatially related patterns, identify heterogeneous units, and suggest spatial states [26]. The calculation of the REL for Fuzhou from 2015 to 2019 based on cold and hot spots shows spatial aggregations of similar values (Fig 3). The initial cold spots appeared in Zhenghe, Zhouning and Shouning in the form of sporadic points, and gradually developed into a blocky clustered distribution. At the same time, Jianning, Taining, Jiangle, Mingxi and other places in the cold spots are distributed in blocks. The sub-cold spots began to appear sporadically around Shunchang, Gutian and Dehua and eventually were concentrated around the cold spot area in 2019.

Hot spots began to gradually and sporadically appear in coastal cities such as Jinjiang, Hui'an, and Nan'an and finally developed into an agglomerated cluster distribution in 2019. The sub-hot spots began to appear in Fuqing, Yongchun, and Xianyou, showing a trend of encircling the hot spot. The comprehensive analysis of the G index spatial distribution for REL shows that the REL of Fujian Province is spatially correlated, with high levels of livability in the southeast and low levels in the northeast.

## Conclusions

The lessons learned from COVID-19 are preliminary because the hazard is an ongoing event. A systematic approach is needed to adapt the concept of ecological sustainability to enhance personal, social and ecological health in the human world. In this paper, we develop a comprehensive multifactor (production-living-ecology) evaluation system to measure the REL of Fujian Province. Our conclusions are as follows.

First, from the temporal perspective, the overall development of the rural ecological livability level in Fujian Province is not large. This may be because construction to improve REL is still being implemented, and no significant effect has yet been seen. Second, from the spatial perspective, REL in Fujian Province is uneven, and spatial correlations are strong. Third, in considering the factors influencing REL and the index layer levels, it can be seen that the value-added of the secondary industry and per-capita annual fiscal revenue in each region are important drivers of REL, while the amount of chemical fertilizer applied and reductions in the ecological afforestation area are important reasons for the decline in the REL level.

Due to the spatial heterogeneity of REL in Fujian Province, this comprehensive evaluation shows that county-level ecological governance also faces challenges such as cross-basin, cross-regional governance and large differences in rural development. County and city governments should strengthen their interregional cooperation and promote the establishment of a platform for unified and synchronized information sharing and monitoring to improve the ecological status of Fujian Province.

This study contributes to the existing literature in the following three ways:

1. To the best of our knowledge, this the first application of the production-living-ecological model, which better evaluates the current situation of Chinese REL, in this field.
2. To a certain extent, the entropy method we used can prevent data loss and lead to more objective decisions, and exploring the factors that influence rural ecological livability can help governors develop guidelines for rural areas to proceed with regeneration plans leading toward more livable districts. Moran's I and Getis-Ord Gi* were used to analyze spatial and temporal differences in and distributional characteristics of the REL level in Fujian.
3. The study of REL in China's most economically important province can provide a reference for the assessment of the ecological environment and rural livability in other developing countries.

With the development of environmentally friendly and resource-preserving rural areas as the goal and the rational and scientific development of the three life functions of production-living-ecology as the principle, this paper emphasizes the optimization of dominant functions, the promotion of medium functions and the improvement of weak functions, thus providing a scientific basis for encouraging the cultivation of rural functions and determining the development direction of rural areas in Fujian Province. The government should guide residents' use of green production technology, green consumption, and green living and should also guide other production, living and consumption methods that need to be made even more green and lower carbon for less ecologically habitable areas. Moreover, the system of an ecologically responsible civilization, natural resource asset audits of leading cadres, evaluation of corporate environmental credit, trades of emissions rights, discounts for environmental pollution discount and other reforms should continue to be explored. Limited by the availability of data (especially of the ecological data for various counties/cities), we collected data from the last 5 years only, which resulted in a short time series and no obvious temporal effect. Follow-up studies should continue to refine the data used.
